# Breaking Data Encryption Standard with a Reduced Number of Rounds Using Metaheuristics Differential Cryptanalysis

**DOI:** 10.3390/e23121697

**Published:** 2021-12-18

**Authors:** Kamil Dworak, Urszula Boryczka

**Affiliations:** Faculty of Science and Technology, University of Silesia in Katowice, Będzińska 39, 41-200 Sosnowiec, Poland; urszula.boryczka@us.edu.pl

**Keywords:** differential cryptanalysis, metaheuristics, symmetric block ciphers, memetic algorithms, DES, simulated annealing

## Abstract

This article presents the author’s own metaheuristic cryptanalytic attack based on the use of differential cryptanalysis (DC) methods and memetic algorithms (MA) that improve the local search process through simulated annealing (SA). The suggested attack will be verified on a set of ciphertexts generated with the well-known DES (data encryption standard) reduced to six rounds. The aim of the attack is to guess the last encryption subkey, for each of the two characteristics Ω. Knowing the last subkey, it is possible to recreate the complete encryption key and thus decrypt the cryptogram. The suggested approach makes it possible to automatically reject solutions (keys) that represent the worst fitness function, owing to which we are able to significantly reduce the attack search space. The memetic algorithm (MASA) created in such a way will be compared with other metaheuristic techniques suggested in literature, in particular, with the genetic algorithm (NGA) and the classical differential cryptanalysis attack, in terms of consumption of memory and time needed to guess the key. The article also investigated the entropy of MASA and NGA attacks.

## 1. Introduction

The growing popularity of computerisation, and at the same time the Internet itself, results in a growing demand for more and more advanced security methods. Restrictions such as individual user access control or basic authentication have become insufficient today. For several decades, engineers concentrating on the topic of information security have designed special cryptographic algorithms that meet the most important security aspects.

The main assumption of cryptography is not to hide the fact of the existence of information, but to keep its real image secret. The message is transformed in such a way that it is readable only to its author and the recipient it is dedicated to [1,2].

Contemporary symmetric block ciphers implement the process of transformation of the plain text using the Feistel cipher and the generalized substitution-permutation network [2]. In 1990, a completely new cryptanalytical method was made public, namely differential cryptanalysis [3]. In the case of the most modern and advanced encryption algorithms, the differential cryptanalysis itself turns out to be ineffective. In order to improve the attack performance, it was proposed to combine metaheuristic algorithms with the differential cryptanalysis algorithm.

In general, metaheuristic algorithms are used to obtain approximate solutions. In the case of cryptanalysis, it is necessary to guess the ideal decryption key—an approximate solution is unacceptable. Due to the avalanche effect present in every encryption algorithm today, changing any bit at the input causes a complete mixing of all bits at the output, which in fact results in the generation of a completely new ciphertext [1]. The developed algorithm enables automatic sifting of the keys with the worst value of the fitness function, owing to which the set of potential solutions will be significantly reduced.

Additional analytical properties of memetic algorithms improve the local search process in such a way as to achieve the best solution in the shortest possible time.

Metaheuristic algorithms are more and more often used in computer science, and thus in the domain of computer security. In the literature, we can find publications describing all kinds of metaheuristic attacks targeting both classical ciphers, contemporary symmetric block ciphers and stream ciphers. A literature review of publications is presented in Table 1.

In [4], the authors focused on evolutionary cryptanalysis using GA on DES4 ciphers by comparing the same bits between original and encrypted ciphertexts. Tadros in [5] presented another GA used to break FEAL8 and DES4 ciphers. Garg in [6] included a comparison between MA and GA during cryptanalysis of SDES encryption algorithm relying on n-gram statistics and frequency analysis method. Another approach was present by Hu in [7], quantum-inspired GA has been applied to break TEA. Abd-Elmonim described another attack, based on the PSO algorithm, responsible to break the full 16-rounded DES cipher in [8]. Vimalathithan and Valarmathi presented their researches about combining the effectiveness of GA and PSO as a new Generic Swarm Optimization algorithm to attack SDES cipher. In 2012, Jadon [10] and Pandey, with Mishra published interesting approaches related to Binary PSO and original PSO algorithms used in cryptanalysis attacks dedicated to DES cipher.

In the following years, Ali [12], Mekhaznia and Menai [14], Bhateja [15], Jain [16,21], and Sabonchi [26] focused on cryptanalysis of classical ciphers such as substitution, transposition, and Vigenere ciphers using many popular metaheuristics like Bees, EA, ACO, PSO and Cuckoo Search algorithms.

Amic in [17,20,28] presented Binary Firefly, Binary Cat Swarm Optimisation (BCSO), and Dolphin Swarm (DSA) algorithm—all directed against DES cipher. In [24] Kamal described the Binary Cuckoo Search algorithm used on ciphertext generated by SDES cipher.

Polak and Boryczka presented new cryptanalysis attacks dedicated to another subset of encryption algorithms—stream ciphers (RC4, VMPC, and RC4+), using Tabu Search in [23,25]. In 2021, Grari [27] published ACO algorithm dedicated Markle-Hellman cipher.

The next chapter is dedicated to a brief introduction to symmetric block ciphers and the DES cipher. The third chapter presents the basic assumptions of differential cryptanalysis, which were used and which constituted a basis for the design work on the MASA algorithm. Chapter four contains a detailed description of the developed metaheuristic attack carried out with the use of MA. The next chapter focuses on describing the runtime environment, including presenting all the parameters selected for each attack. This chapter also presents the results of the experiments, including the entropy studies for the MASA and NGA algorithms. The second to last chapter presents a detailed analysis of the effectiveness of the attacks presented, both in terms of the number of proven solutions and the time of decryption of the cryptogram. The article is concluded with a brief summary of the various stages of the research. This chapter also suggests further research directions. Appendix A is attached to this article, detailing the results for the $\Omega_{2}$ characteristic.

## 2. Symmetric Block Ciphers

Symmetric ciphers are still one of the most popular encryption algorithms. In this type of ciphers, only one main key is used, which simultaneously takes on a role of an encryption and decryption key, which can be written as $K_{E}=K_{D}$. In the case of block ciphers, each message is divided into a finite number of blocks of the same length—for example, 64-bit blocks. Then they are transferred to the appropriate encryption function. Exactly one block of the ciphertext is generated from one block of plain text. If the message cannot be divided into even blocks, an additional block is created to store the last, incomplete, fragment of data. Then, for consistency, it is supplemented with default values or zeros.

These algorithms are perfect for encrypting larger volumes of data stored, that is, in all kinds of warehouses, wholesalers or databases. The most popular block cipher schemes include ciphers such as: DES and AES.

### Data Encryption Standard

The DES cipher has been designed in such a way that the avalanche effect occurs from the very beginning of the algorithm [1]. Changing any input bit forces us to change at least half, and sometimes even all, of the output bits. The state of each bit at the output depends on each bit specified at the input [29].

**Figure 1:**
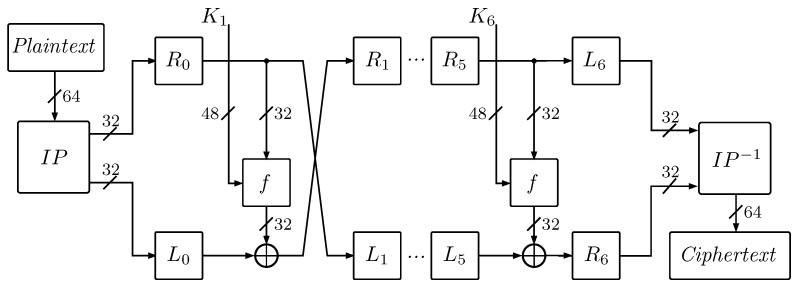
Simplified diagram of the six-rounded Data Encryption Standard DES algorithm.

The basic version of the cipher converts 64-bit plain text blocks into 64-bit ciphertext blocks, using a 64-bit encryption key *K*  [2,30]. After running the algorithm, the primary key is reduced to 56 bits by removing every eighth parity bit. *K* is then subjected to breaking into six 48-bit subkeys, used in each of the cipher rounds, $K_{1},...,K_{6}$—A description of the primary key distribution process is presented in detail in [1,2,29,30,31,32]. Figure 1 shows a 6-round DES algorithm.

The plain text block is passed to the initial IP permutation. Then, the generated block is divided into two regular 32-bit parts, *R* and *L*. In the next steps, six identical encryption cycles will be run, in which the right part of the $R_{i}$ is passed to the *f*-round function along with the corresponding subkey $K_{i}$. Then, the generated data block is subjected to the exclusive disjunction operation with the left part of the $L_{i}$, resulting in a new right part of the $R_{i+1}$. The new left part of the $L_{i+1}$ is copied from the right part of the previous $R_{i}$ cycle.

After all the cipher rounds have been completed, parts of the $L_{6}$ and $R_{6}$ are combined into a 64-bit block, which will undergo the last transformation by the $IP^{-1}$ inverse permutation function. The result of transposition of individual bits will be a 64-bit cryptogram block.

**Figure 2:**
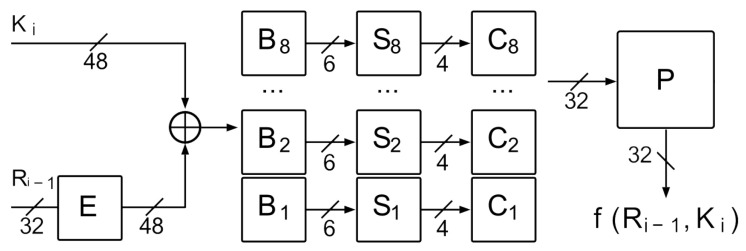
The *f* round function of the Data Encryption Standard DES algorithm. The only one, nonlinear, element of the DES cipher.

The *f* round function has been visualized in Figure 2. As an input parameter, a 32-bit data block is given, which at the very beginning will be extended via permutation *E*. The aim of this transformation is to align the length of the transferred block with the size of the subkey by duplicating the selected bits. By allowing one bit to influence two substitutions, the avalanche effect is increased [1]. The generated sequence is modulo two sum with subkey bits and then divided into eight 6-bit $B_{1}$–$B_{8}$ blocks.

Each of the $B_{j}$ blocks will be transferred to the so-called substitution matrix called S-blocks $S_{j}$. The main aim of this transform is to compress the input data. 6-bit data blocks will be converted into 4-bit blocks. $S_{j}$ consist of integers between 0 and 15, stored in matrices of sixteen columns and four rows. The first and last bits of a 6-bit sequence $B_{j}$ determine the line number. The remaining four bits represent the number of the column from which the return value will be selected [1,2,30].

$S_{j}$ are the only nonlinear element of the DES standard. Changing one bit in an input sequence can lead to a complete mixing of all generated bits at the output. Modifications carried out in them have a significant impact on the level of complexity of cryptanalysis of the entire cipher. At the end of the *f* function, the generated sequences are combined into one 32-bit block, which will be passed to the permutation *P*—aimed at mapping each of the input bits to exactly one output bit without duplicating or omitting any of them [1].

## 3. Differential Cryptanalysis

The suggested algorithm is based on an attack with selected plain text. At the beginning, it should be assumed that the cryptanalyst has continuous access to the encryption algorithm, which allows him to select a pair of plain texts and analyse the generated ciphertexts. It is important that the tested pairs must differ from each other in a certain way. Most symmetric block ciphers determine this difference on the basis of a simple symmetric difference operation, which is written as $P^{\prime }=P⊕P^{*}$, where *P* and $P^{*}$ are two crafted plain texts. Pairs may be generated in a pseudorandom way, although the most important condition is the difference $P^{\prime }$, which must follow the established process. Next, the cryptanalyst checks how the determined difference changes in the subsequent phases of the cipher. Using the difference between the texts in individual iterations of the cipher, for a sufficiently large number of pairs, it is possible to assign different probabilities, suggesting the correctness of some subkeys [3]. When analyzing subsequent pairs of plain texts and ciphertexts, it turns out that one key may be more probable than the others.

Every modern cipher is non-linear—it means that it is not possible to find any pattern or rule by which to determine the value of a function for the next argument [3]. This nonlinearity is obtained via the round *f* function. Each of all possible differences is characterized by a certain probability, which determines how often the *f* function returns the expected value [3]. These differences are called characteristics Ω. All possible characteristics can be determined by means of an additional matrix, where the rows correspond to all possible symmetric differences of the input blocks, and the columns to all possible symmetric differences of the output blocks [1]. Each of the elements will determine how many times the sum of the output bits occurs for the selected sum of the input bits.

By analysing the diagram shown in Figure 2, the input symmetric difference $B^{\prime }$ can be determined assuming that $E=E(R_{i-1})$:

(1) $$B^{\prime }=\overset{8}{\underset{j=1}{∥}}B_{j}⊕B_{j}^{*}=\overset{8}{\underset{j=1}{∥}}\left(E_{j}\left(R_{i}\right)⊕K_{i}\right)⊕\left(E_{j}\left(R_{i}^{*}\right)⊕K_{i}\right)=\overset{8}{\underset{j=1}{∥}}E_{j}⊕E_{j}^{*},$$

where symbol ∥ stands for the concatenation of the successive data blocks. From the expression above, it can be seen that $B^{\prime }$ has nothing to do with the subkey. When the value of each $B_{j}^{\prime }$ is known, the set of all ordered pairs $\left(B_{j},B_{j}^{*}\right)$ can be determined for the input symmetric difference as suggested in [31]:

(2) $$\Delta \left(B_{j}^{\prime }\right)=\left\{\left(B_{j},B_{j}⊕B_{j}^{\prime }\right):B_{j}\in {\left(Z_{2}\right)}^{6}\right\}.$$

Knowing the output difference $C_{j}^{\prime }=S_{j}\left(B_{j}\right)⊕S_{j}\left(B_{j}^{*}\right)$, it becomes possible to generate the distribution of all possible input differences to all output differences according to the theorem described in [31]:

(3) $$IN_{j}\left(B_{j}^{\prime },C_{j}^{\prime }\right)=\left\{B_{j}\in {\left(Z_{2}\right)}^{6}:S_{j}\left(B_{j}\right)⊕S_{j}\left(B_{j}⊕B_{j}^{\prime }\right)=C_{j}^{\prime }\right\}.$$

Most often, this distribution will be steady. The cryptanalyst’s task is to find distributions that are as unsteady as possible. Based on the expression (3), an additional test set can be determined using the following formula [31]:

(4) $$test_{j}\left(E_{j},E_{j}^{*},C_{j}^{\prime }\right)=\left\{B_{j}⊕E_{j}:B_{j}\in IN_{j}\left(E_{j}^{\prime },C_{j}^{\prime }\right)\right\}.$$

If the number of elements in $test_{j}$ is equal to the power of $IN_{j}$ set, then the set must contain bits of the $K_{ij}$ subkey [31].

This method makes it possible to restore the correct decryption key using $2^{47}$ selected plain texts and the corresponding ciphertexts.

## 4. Metaheuristics Differential Cryptanalysis

**Figure 3:**
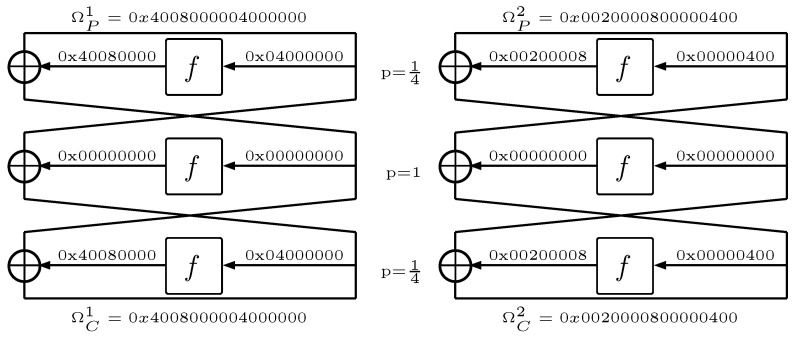
The two the most probable 3-round characteristics $\Omega_{P}^{1}$ and $\Omega_{P}^{2}$ for six rounded cipher DES [31,32].

From the point of view of the developed attack, the IP and $IP^{-1}$ permutations may be omitted. The algorithm begins by selecting the two most probable 3-round characteristics $\Omega_{P}^{1}$ and $\Omega_{P}^{2}$ mentioned in [31,32], which are presented in Figure 3, where *P* denotes characteristics for plaintext and *C* for ciphertexts.

The probability of each characteristic is exactly $P_{\Omega }=\frac{1}{16}$ in the fourth round of the encryption algorithm S-Blocks $S_{2},S_{5},S_{6},S_{7},S_{8}$ for $\Omega_{P}^{1}$ and $S_{1},S_{2},S_{4},S_{5},S_{6}$ for $\Omega_{P}^{2}$ for some input symmetric difference $B_{j}^{\prime }$ return an output symmetric difference $C_{j}^{\prime }$ equal to zero. Owing to this, it becomes possible to determine the sets $I_{1}=\left\{2,5,6,7,8\right\}$ for $\Omega_{P}^{1}$ and $I_{2}=\left\{1,2,4,5,6\right\}$ for $\Omega_{P}^{2}$. The further description of the attack is identical for each of the characteristics Ω so it was decided to generalize it by introducing one generic *I* set consisting of elements of sets $I_{1}$ and $I_{2}$.

The next step will be to generate a set of plain text pairs, along with a set of corresponding cryptograms, where the symmetrical difference will correspond to the characteristics $\Omega_{1}$ and $\Omega_{2}$. The number of pairs needed is calculated using the signal-to-noise ratio [3]:

(5) $$S/N=\frac{m\cdot p}{m\cdot \alpha \cdot \beta /2^{k}}=\frac{2^{k}\cdot p}{\alpha \cdot \beta }=\frac{2^{30}\cdot {}^{1}{/}_{16}}{4^{5}}=2^{16},$$

where:

- *m*—the number of pairs generated, having no effect on S/N;
- *p*—the probability of the selected characteristic Ω;
- *k*—number of bits of the subkey;
- α—the average number of subkeys, suggested by one pair;
- β—the ratio of the analysed pairs to all possible ones.

As suggested in [3], for $S/N=2^{16}$, 7–8 correct pairs are needed for each of the characteristics. Due to the probability of $P_{\Omega }$, a minimum of 150–200 pairs of plain text should be generated [3].

Additionally, the $test_{j}$ test set is determined, owing to which it will be possible to partially filter pairs from the set. If the power of the test set for at least one element from set *I* is equal to 0, the pair may be rejected:

(6) $$\underset{j\in I}{⋀}\left|test_{j}\right|>0.$$

The aim of the suggested attack is to guess the last $K_{6}$ encryption subkey. If the difference of $C^{\prime }$ and part of $R_{5}$ is known, it becomes possible to analyze the various subkeys closely by comparing all bits of the output of the S-blocks with $C^{\prime }$. A brute-force attack would need to check all $2^{30}$ solutions. MA can be used as an optimization tool that finds the correct solution in much shorter time.

Each individual is represented by a 30-bit $K_{j}$ subkey. The fitness function is defined with the following formula:

(7) $$F_{f}=\sum_{i=0}^{n}L-\sum_{j\in I}H\left(\left(S_{j}\left(B_{j}\right)⊕S_{j}\left(B_{j}^{*}\right)\right),P^{-1}\left(R_{6}^{\prime }⊕L_{3}^{\prime }\right)\right),$$

where:

- *H*—is the Hamming distance;
- *L*—the length of the subkey.

Owing to the knowledge of the probability of $P_{\Omega }$, it is possible to estimate the value of $L_{3}^{\prime }$, while $R_{6}^{\prime }$ can be obtained by analyzing a pair of generated ciphertexts. $F_{f}$ counts the number of overlapping bits between the difference obtained from the S-blocks and the $C^{\prime }$ difference.

The algorithm uses standard one-point crossover. The locus is selected pseudorandomly from 1 to 30. The newly created subkeys can be modified with the use of a mutation operator—which consists in replacing two pseudorandomly selected bits. The algorithm selects individuals using tournament selection. A leader is elected from the set of all subkeys and it is passed to the crossover operator.

There is an additional local search process in the algorithm—it is performed using the simulated annealing algorithm. The MASA attack pseudocode for the ΩP characteristic is shown below. Due to the complexity of this algorithm, it was decided to divide it into two parts:

- the first one, Algorithm 1—responsible for generating a set of filtered pairs of plain text, ciphertexts and determining the $test_{j}$ test set for each of the indexes;
- the second one, presented in Algorithm 2—describing the memetic algorithm, along with the processes of selection, crossing, mutation and exploitation, taking into account the pseudocode of the basic simulated annealing algorithm.

**Algorithm 1:** The pseudocode of the set of pairs preparation process for the MASA attack.
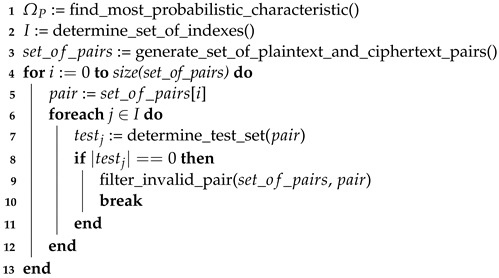


Running the MASA algorithm for $\Omega_{P}^{1}$ will make it possible to guess 30 out of 48 bits of the $K_{6}$ subkey. Re-running the algorithm, this time for $\Omega_{P}^{2}$, allows us to find an extra 12 bits. In order to obtain the remaining 6 bits of the last $K_{6}$ subkey—coming from the S-block $S_{3}$, we can use the brute-force method. Having the $K_{6}$ subkey, it is possible to recover 48 out of 56 bits of the decryption key by reversing the key decomposition process. The remaining 8 bits can be guessed using the brute-force method once again—for example, a brute force attack.
**Algorithm 2:** MASA attack pseudocode.
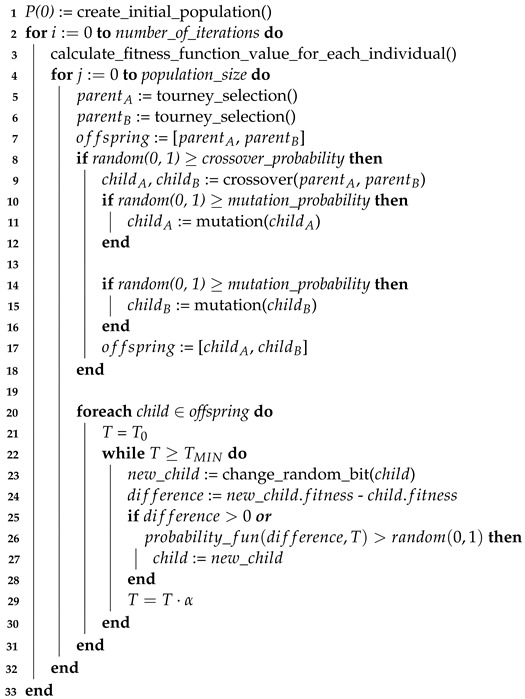


## 5. Experimental Results

This chapter describes the analysis of the proposed memetic attack MASA and NGA in terms of the quality and number of solutions obtained [22]. It was important to check whether the suggested algorithms make it possible to improve the time of finding the correct subkey. Another important aspect was to check whether the MASA memetic algorithm enables a more effective, and therefore more successful, differential cryptanalysis.

### 5.1. Selecting Parameters

As part of the experiments, the impact of the parameters listed below for each of the attacks on the convergence of the algorithm and the quality of the obtained solutions was examined:

- number of iterations for the MASA and NGA algorithms;
- population size for the MASA i NGA algorithms;
- number of plaintext and ciphertext pairs γ for the MASA and NGA algorithms;
- probability of the heuristic negation $P_{n}$ for the NGA algorithm.

In the conducted experiments, the parameter values were used in various combinations and for the subsequent experiments, potentially the best values in terms of the running time of the algorithm were established. For the MASA memetic algorithm, the parameters were set according to Table 2 below:

The description of the NGA algorithm parameters has been described in detail in the publication [19]. Table 3 presents the most important parameters of the NGA algorithm:

As was mentioned before, for the purposes of the tests, a simplified version of the DES cipher was used, in which the number of rounds was limited from 16 to 6. All other processes in the encryption algorithm, such as subkey generation and S-block compression, remained unchanged.

### 5.2. Comparative Study

Each of the algorithms was tested 30 times for each of the characteristics Ω. Table 4 below shows the value of the $F_{f}$ fitness function for the MASA and NGA algorithms for the first characteristic $\Omega_{1}$. The remaining results—for the characteristic $\Omega_{2}$ are given in Appendix A in the Table A1.

Experiments in which the correct decryption key could not be guessed were marked in bold in the table above.

The probability of each of the characteristics for this cipher is not 100%. It means that despite striving for the maximum value of the fitness function, it will never be achieved. The inability to obtain the maximum value means that we are not able to terminate the running of the algorithm earlier than after the completion of all predetermined iterations.

**Figure 4:**
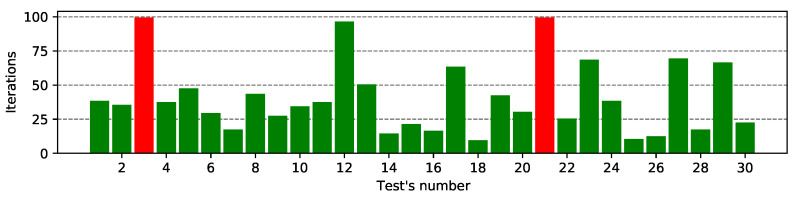
List of correctly guessed bits of MASA attack for the $\Omega_{1}$ characteristic.

**Figure 5:**
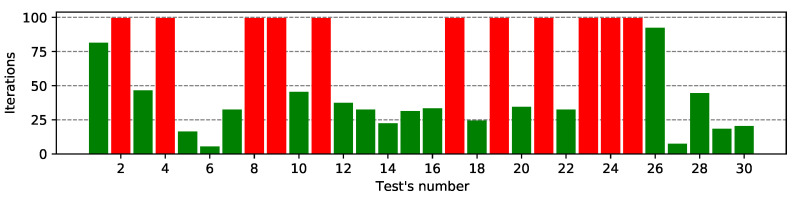
List of correctly guessed bits of NGA attack for the $\Omega_{1}$ characteristic.

**Figure A1:**
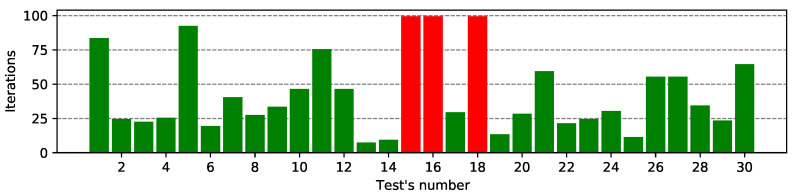
List of correctly guessed bits of MASA attack for the $\Omega_{2}$.

**Figure A2:**
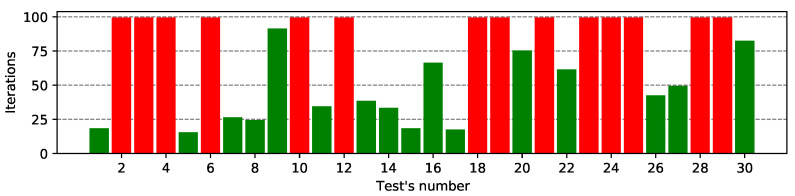
List of correctly guessed bits of NGA attack for the $\Omega_{2}$.

Figure 4 and Figure 5 present a list of all correctly guessed bits of the $K_{6}$ subkey for the MASA and NGA algorithms for the $\Omega_{1}$ characteristic. The remaining results—for the $\Omega_{2}$ characteristic are present in Appendix A in the Figure A1 and Figure A2.

In a large number of cases, the MASA attack finds the correct subkey in the first 25 iterations. In approximately 6–7 cases, the algorithm found a solution using half of the available iterations, while in the other two cases (tests #3 and #21, marked as red on the figure) the attack failed to cope with the given ciphertext. The algorithm found the correct decryption subkey in 93% of the cases - markes as green on the figure.

In the case of the NGA algorithm, the cipher was not cracked 11 times—which is over 37% of all possible approaches—red bars on the figure. During the remaining 63% of the tests, it was possible to crack the cipher with the decryption algorithm—green color. In most cases, it was possible to guess the correct subkey using only 30–40 iterations. The tests with identifiers #1 and #26 also deserve special attention. They show a very large number of iterations (over 80), which means that the NGA algorithm found the correct solution at the very end of its running.

On the presented bar plots we can notice the MASA algorithm is much effective because it successfully found the correct subkey in almost every test when NGA attack has worked in only 63% of experiments. Simulated annealing, used as an additional exploitation step of the MA, is more effective than the heuristic negation operator used in the NGA attack.

**Figure 6:**
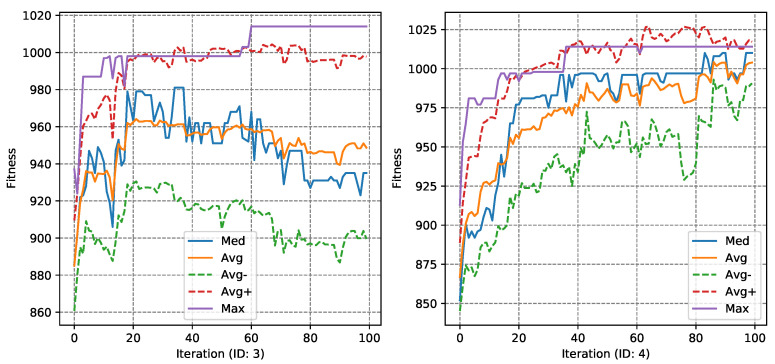
The MASA fitness function $F_{f}$ convergence diagrams for $\Omega_{1}$ (tests #3 and #4).

**Figure 7:**
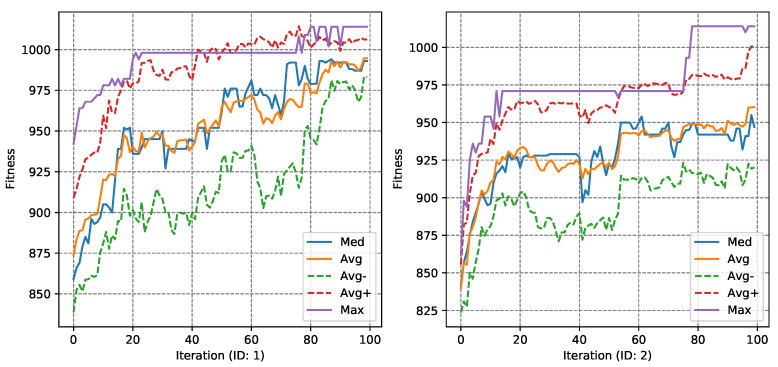
The NGA fitness function $F_{f}$ convergence diagrams for $\Omega_{1}$ (tests #1 and #2).

**Figure A3:**
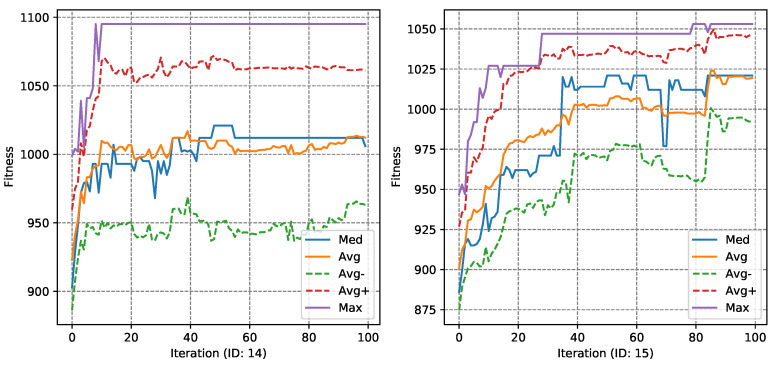
The MASA fitness function $F_{f}$ convergence diagrams for $\Omega_{2}$ (tests #14 and #15).

**Figure A4:**
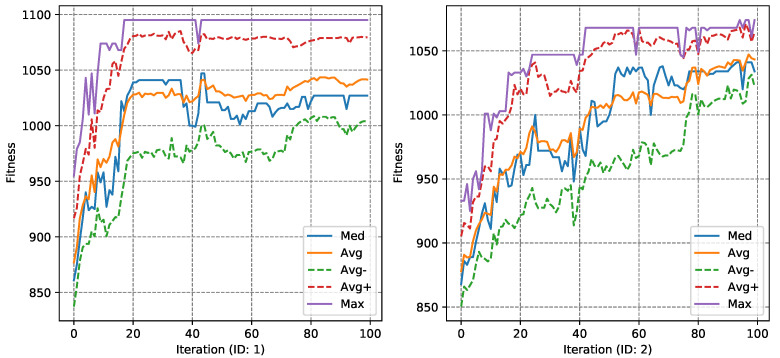
The NGA fitness function $F_{f}$ convergence diagrams for $\Omega_{2}$ (tests #1 and #2).

The next stage of the experiments was to analyze the course of the fitness function value using the convergence diagrams, which were presented successively, for the MASA attack and $\Omega_{1}$ in Figure 6 and Figure 7, for the NGA algorithm. Convergence diagrams for the $\Omega_{2}$ were present in Appendix A in the Figure A3, for the MASA algorithm, and Figure A4 for the NGA attack.

The above graph shows tests #3 and #4 with minimum, maximum, medians and averages—and average values increased and decreased by the standard deviation of the fitness function. The tests were selected in such a way as to visualize both a positive case—when it was possible to guess the correct subkey, and a negative one.

In the case of both tests of the MASA algorithm, a rapid increase in the maximum value of $F_{f}$ can be noticed at the very beginning of the algorithm’s running. In further iterations, there are single drops of this value, after which the maximum value is stabilized and then increased again. The median for 60% of the algorithm’s running time remains similar, only at the very end of its running we can notice its decrease. When analyzing the case #4 diagram, already in the first iterations of the algorithm, a rapid increase in the median value can be observed—the majority of individuals in the population have a similar value of the fitness function. This may be related to the algorithm falling into the local extreme, which it has not managed to leave.

**Figure 8:**
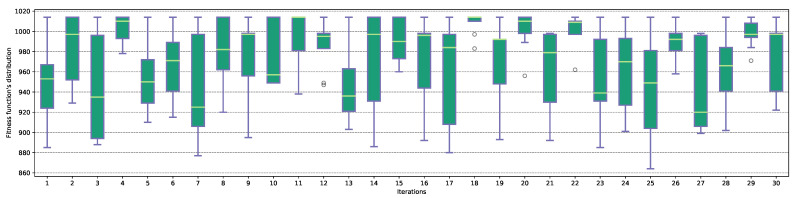
The distribution of the fitness function $F_{f}$ values in the last iteration for the MASA algorithm and $\Omega_{1}$ characteristic.

**Figure 9:**
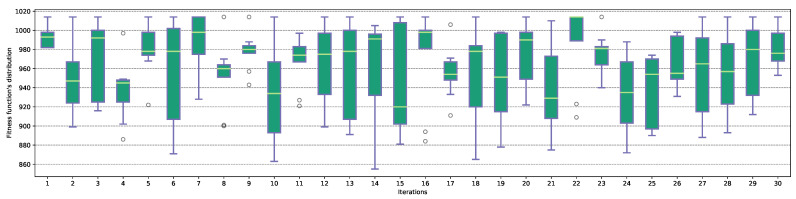
The distribution of the fitness function $F_{f}$ values in the last iteration for the NGA algorithm and $\Omega_{1}$ characteristic.

**Figure A5:**
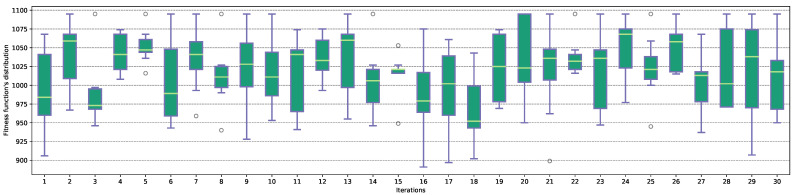
The distribution of the fitness function $F_{f}$ values in the last iteration for the MASA algorithm and $\Omega_{2}$ characteristic.

**Figure A6:**
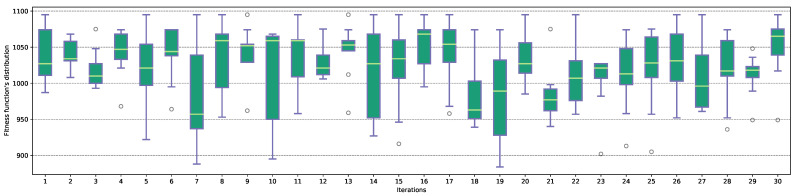
The distribution of the fitness function $F_{f}$ values in the last iteration for the NGA algorithm and $\Omega_{2}$ characteristic.

The next stage of the tests was to review the distribution of the fitness function values in the last iteration of each attack—the distribution is presented in Figure 8 for the MASA algorithm, and Figure 9 in the case of an NGA attack. Boxplots for the $\Omega_{2}$ characteristic were present in Appendix A in the Figure A5, for the MASA algorithm, and Figure A6 for the NGA attack.

In the case of the MASA algorithm, some of the tests—for example, #18, #20 or #22—are characterized by a high degree of homogeneity, which means that the population is characterized by a low diversity of individuals. When analyzing each of the attacks, a large degree of variability between individuals can be observed, which is undoubtedly indicated by the median value, changing its position between the first and the third quartiles. In the case of the NGA algorithm, in some experimentes, an unexpected increase of the value of the fitness function can be observed at the very end of the algorithm’s running—it is evidenced by the presence of the outlier of the maximum value.

The MASA and NGA attacks are characterized by a certain degree of pseudo-randomness. In order to perform statistical verification of the algorithms, a non-parametric Wilcoxon’s test was used to compare the results. The hypothesis $H_{0}$, specifying no difference when comparing the samples, and the hypothesis $H_{1}$, assuming a difference between the two samples, were set. The following criteria were used to perform the test:

- value of the fitness function—performed for the best quality subkeys found for each run;
- number of subkeys checked.

The weight of each criterion was expressed at the same value, set to 0.5. For the analyses performed, hypothesis $H_{0}$ was rejected at *p* < 0.05—thus indicating the statistically important differences between the best results retrieved. The results obtained through the MASA algorithm are significantly better than the NGA attack.

### 5.3. Entropy Study

The possibility to maintain a highly diverse population may improve the algorithm’s ability not to fall into local extremes. In order to estimate the size of the disorder in the system, the entropy was used:

(8) $$H\left(X\right)=\sum_{i=1}^{n}p\left(x_{i}\right)log_{2}\frac{1}{p(x_{i})}=-\sum_{i=1}^{n}p\left(x_{i}\right)log_{2}p\left(x_{i}\right).$$

The entropy was computed by comparing the respective bits of each subkey with the corresponding bits of the best-adapted individual. An example for the population P={11101,10101,11011,11110}, where the last individual 11110 is the leader, is presented below (Table 5):

where:

- $p(x_{1})$—the probability of an identical bit occurring in a given position between individuals and the leader;
- $p(x_{2})$—the probability of a different bit occurring in a given position between individuals and the leader;
- $H(x_{1})$—entropy values for the probability $p(x_{1})$, at a given position;
- $H(x_{2})$—entropy values for the probability $p(x_{2})$, at a given position.

Based on the example listed in Table 5, the entropy value of the entire system can be computed as follows:

(9) H=−(0+0−0.93−0.5−0.93−0.5−1−1−0.5−0.93)=6.29.

**Figure 10:**
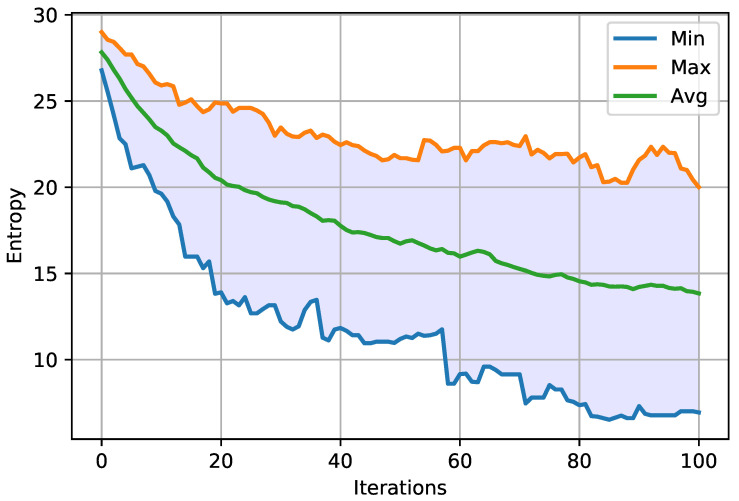
Minimum, maximum and average entropy, during all iterations, for MASA algorithm and $\Omega_{1}$ characteristic.

**Figure 11:**
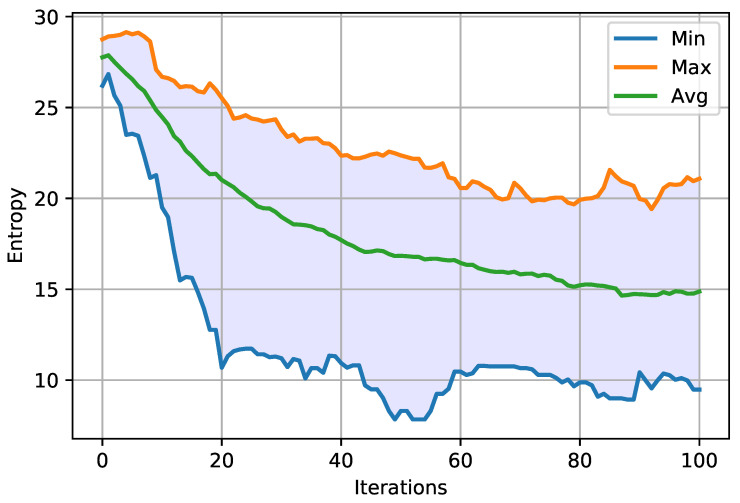
Minimum, maximum and average entropy, during all iterations, for NGA algorithm and $\Omega_{1}$ characteristic.

**Figure 12:**
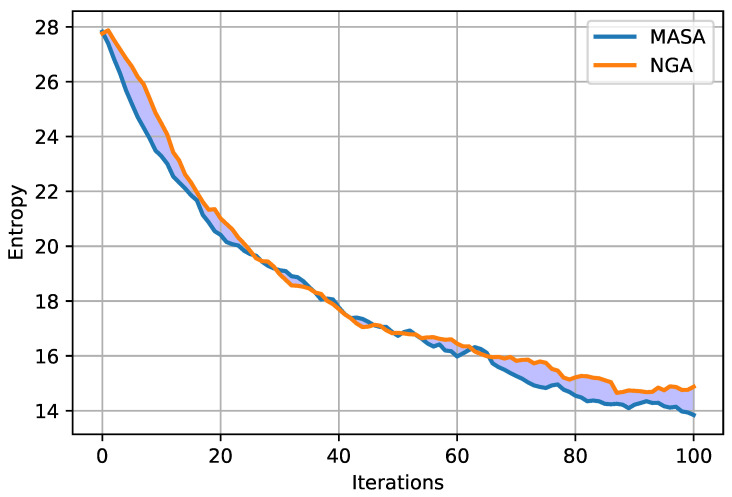
The comparison of the entropy of the MASA and NGA algorithms for the $\Omega_{1}$ characteristic.

**Figure A7:**
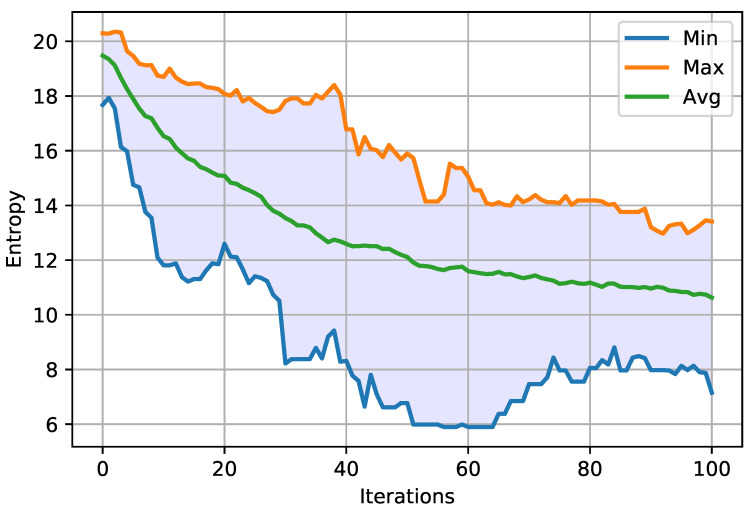
Minimum, maximum and average entropy, during all iterations, for MASA algorithm and $\Omega_{2}$ characteristic.

**Figure A8:**
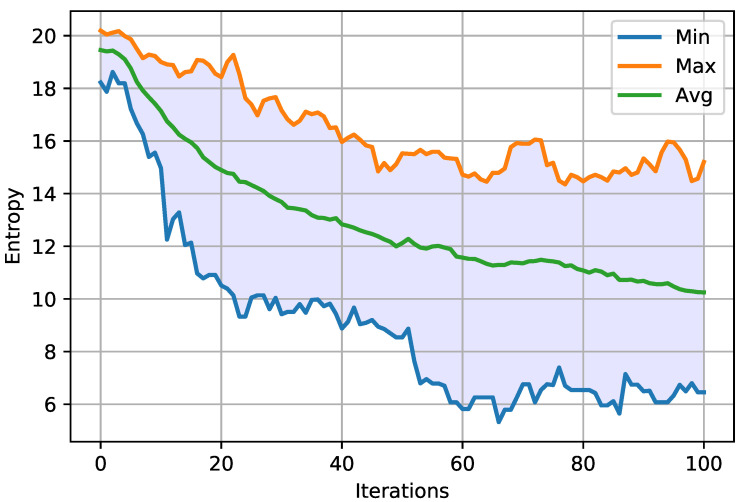
Minimum, maximum and average entropy, during all iterations, for NGA algorithm and $\Omega_{2}$ characteristic.

**Figure A9:**
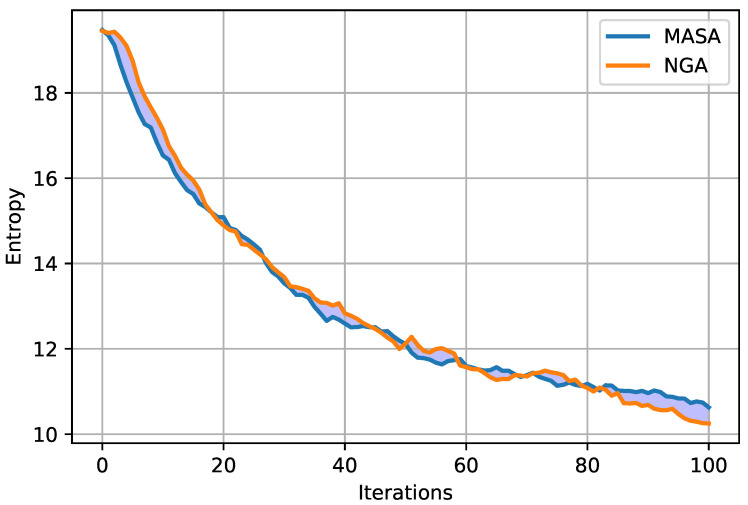
The comparsion of the entropy of the MASA and NGA algorithms for the $\Omega_{2}$ characteristic.

Entropy for the MASA and NGA algorithms was visualized respectively in Figure 10 and Figure 11. The charts show the maximum, minimum and average values. Moreover, it was decided to visualize the average value of entropy for both attacks on one graph, which is presented in Figure 12. The remaining results—for the characteristic $\Omega_{2}$ are given in Appendix A in Figure A7, Figure A8, Figure A9. 

The entropy value was computed during each iteration and 30 launches of MASA and NGA attacks. During all the conducted tests, identical pairs of plain text and the corresponding ciphertexts were used, as well as the same encryption key—owing to which it was possible to make the most reliable comparison.

When analyzing the graphs presented above (Figure 10 and Figure 11), a decrease in the entropy value can be noticed from the very beginning of the running of each of the algorithms. In the last iterations, a gradual stabilization of the system becomes visible, which would most probably be more noticeable after increasing the number of iterations. Comparing the average courses, it can be noticed in Figure 12 that the entropy value for the MASA attack is lower from the very beginning. Only from about the thirtieth iteration, the NGA algorithm obtains a similar value, and sometimes even lower, in relation to the MASA attack. Eventually, the entropy values for the NGA algorithm begin to stabilize at around the sixtieth iteration, while in the case of the MASA attack it continues to decrease. At the end of the algorithms’ running, the difference in entropy value between attacks becomes visible.

The experiments carried out and described above clearly confirm the effectiveness of the suggested MASA attack, based on the use of memetic algorithms and simulated annealing. This information may be important during the running of the algorithm, since the probability of leaving the local extremum will be higher, and thus the quality of the final results will be better.

## 6. Conclusions

The article presents the results for the NGA genetic algorithm enriched with an additional heuristic negation operator and the MASA memetic algorithm that performs the local search process through simulated annealing. Both algorithms undoubtedly improve the process of an attack of differential cryptanalysis against the ciphertexts generated with the DES standard. An important aspect is the attempt to minimize the number of verified subkeys, which is presented in the table below:

The developed algorithms improve the effectiveness and efficiency of the attack, which is extremely important from the point of view of a cryptanalyst. Presented metaheuristics cryptanalysis, based on the differential cryptanalysis approach, can be helpful to raise the security level in already implemented IT systems. It can also be used to improve the complexity of ciphers at the design level. Proposed attacks, verified on the DES cipher, can be tested on more complicated modern encryption algorithms like AES or GOST ciphers.

Based on the tests presented in the previous section and Table 6, it is possible to clearly state the superiority of the MASA attack and the NGA algorithm over the classic differential cryptanalysis attack, due to the frequency of correctly guessed subkey and the number of proven solutions.

There are many parameters that influence the quality of offered solutions. Analyzing the importance of individual parameters, we intend in the future to conduct an analysis based on removing some of them or replacing them with a simplified version, without losing the quality of the offered solutions. Such approach (an ablation study) is very common when estimating costs of deep learning solutions and we hope that it will also be very effective here.

Work is currently underway on modifications of the developed attack, which would enable an even faster exploration of the solution space. In the future, an adaptive version of the memetic algorithm is expected to be developed to automatically adjust the attack parameters. A parallel implementation is also planned, which should be much more effective.

Simplified and the original DES encryption algorithms are commonly used by many cryptanalysts as a starting point to perform research and experimental studies in this discipline of science. It can be found in the literature review, presented in Table 1, in the introduction section. The authors of this article decided to use a reduced DES cipher for the purposes of developing new metaheuristic attacks described in the paper. Starting experiments from modern ciphers could be too complicated and significantly extend the research process. At the current state, we can test the proposed algorithms against more advanced symmetric block ciphers such as Twofish, AES, or GOST, which will definitely be the next step in future works.

## Figures and Tables

**Table 1:** Literature review of researches on metaheuristics cryptanalysis.

Year	Authors	Algorithm	Cipher
2007	Song et al. [4]	GA	Four-Round DES
2007	Tadros et al. [5]	GA	Four-Rounded DES
2009	Garg [6]	GA and MA	Simplified Data Encryption Standard (SDES)
2010	Hu [7]	GA	Tiny Encryption Algorithm (TEA)
2011	Abd-Elmonim [8]	PSO	DES
2011	Vimalathithan and Valarmathi [9]	GA, PSO and Genetic Swarm Optimization (GSO)	Simplified Data Encryption Standard (SDES)
2012	Jadon et al. [10]	Binary PSO	DES
2012	Pandey and Mishra [11]	PSO	DES
2013	Ali [12]	Bees algorithm	Substitution Ciphers
2014	Boryczka and Dworak [13]	EA	Transposition Cipher
2014	Mekhaznia and Menai [14]	ACO and PSO	Feistel, Vigenere, and substitution ciphers
2015	Bhateja et al. [15]	Cuckoo Search	Vigenere cipher
2015	Jain et al. [16]	Cuckoo Search	Substitution Ciphers
2016	Amic et al. [17]	Binary Firefly Algorithm	DES
2016	Dworak et al. [18]	GA and MA	Simplified Data Encryption Standard (SDES)
2016	Dworak and Boryczka [19]	EA	Four-Rounded Fast Data Encipherment Algorithm (FEAL)
2017	Amic et al. [20]	Binary Cat Swarm Optimization (BCSO)	DES
2017	Jain et al. [21]	Cuckoo Search	Knapsack Cryptosystem
2017	Dworak and Boryczka [22]	GA	Six-Rounded DES
2018	Polak and Boryczka [23]	Tabu Search	RC4 and VMPC
2019	Amic et al. [20]	Dolphin Swarm Algorithm (DSA)	DES
2019	Kamal et al. [24]	Binary Cuckoo Search	Simplified Data Encryption Standard (SDES)
2019	Polak and Boryczka [25]	Tabu Search	RC4+
2020	Sabonchi et al. [26]	DE, GA and PSO	Vigenere cipher
2021	Grari et al. [27]	ACO	Merkle-Hellman cipher

**Table 2:** Parameters of the MASA algorithm.

Id	Parameter	Symbol	Value
1	Maximum number of iterations	$It_{MAX}$	100
2	Population size	*N*	10
3	Number of plaintext pairs	γ	200
4	Tourney size	$T_{SIZE}$	10
5	Crossover probability	$P_{c}$	0.9
6	Mutation probability	$P_{m}$	0.02
7	Initial temperature	$T_{0}$	1
8	Minimal temperature	$T_{MIN}$	0.1
9	Cooling rate	α	0.9

**Table 3:** Parameters of the NGA algorithm.

Id	Nazwa	Symbol	Value
1	Maximum number of iterations	$It_{MAX}$	100
2	Population size	*N*	10
3	Number of plaintext pairs	γ	200
4	Tourney size	$T_{SIZE}$	10
5	Crossover probability	$P_{c}$	0.9
6	Mutation probability	$P_{m}$	0.02
7	Heuristic operator probability	$P_{n}$	0.25

**Table 4:** Fitness function values for MASA and NGA algorithms for characteristic $\Omega_{P}^{1}$.

ID	MASA					NGA				
	Min	Med	Avg	Max	Std. Dev.	Min	Med	Avg	Max	Std. Dev.
1	885	953	95.5	1014	39.9	982	993	994.7	1014	11.5
2	929	997	989.2	1014	30.8	**899 **	**947**	**960.3**	**1012**	**40.3**
3	**888**	**935**	**948.7**	**1012**	**49.2**	916	992	977.4	1014	37.8
4	978	1010	1003.9	1014	12.3	**886**	**945**	**943.0**	**997**	**33.5**
5	910	950	960.9	1014	38.9	922	978	982.3	1014	25.5
6	915	971	971.6	1014	35.1	871	978	960.1	1014	53.2
7	877	925	953.7	1014	52.6	928	998	990.1	1014	30.3
8	920	982	983.6	1014	31.1	**900**	**960**	**958.9**	**1012**	**36.2**
9	895	997	978.6	1014	35.2	**943**	**980**	**981.3**	**1012**	**20.7**
10	949	957	981.0	1014	30.1	863	934	945.9	1014	50.5
11	938	1014	996.8	1014	25.5	**921**	**974**	**973.0**	**997**	**27.6**
12	947	995	988.6	1014	22.1	899	975	965.1	1014	42.1
13	903	936	952.0	1014	36.9	891	978	962.3	1014	48.7
14	886	997	975.5	1014	46.6	855	991	958.0	1014	55.8
15	960	990	992.3	1014	20.0	881	920	951.3	1014	52.3
16	892	996	970.6	1014	42.3	884	998	978.0	1014	45.4
17	880	984	960.8	1014	50.1	**911**	**954**	**962.5**	**1012**	**29.2**
18	983	1014	1008.8	1014	10.0	865	978	958.1	1014	54.5
19	893	992	975.8	1014	38.1	**878**	**951**	**951.3**	**998**	**44.0**
20	956	1010	1003.3	1014	17.7	922	990	977.3	1014	34.1
21	**892**	**979**	**965.9**	**998**	**38.3**	**875**	**929**	**945.5**	**1010**	**45.3**
22	962	1009	1003.6	1014	15.1	909	1014	990.2	1014	38.2
23	885	939	960.7	1014	41.2	**940**	**981**	**978.3**	**1010**	**23.2**
24	901	970	962.2	1014	40.6	**872**	**935**	**936.3**	**988**	**41.9**
25	864	949	949.9	1014	50.3	**890**	**954**	**944.9**	**988**	**34.9**
26	958	992	991.8	1014	15.9	931	955	972.2	1014	28.4
27	899	920	949.2	1014	44.8	888	965	964.5	1014	43.2
28	902	966	965.4	1014	40.2	893	957	959.4	1014	40.6
29	971	997	999.2	1014	13.0	912	980	973.1	1014	37.2
30	922	997	977.2	1014	36.0	953	976	987.7	1014	21.2

**Table 5:** Example scenario of the entropy calculation.

Subkey	Bit 1	Bit 2	Bit 3	Bit 4	Bit 5
A	1	1	1	0	1
B	1	0	1	0	1
C	1	1	0	1	1
Leader	1	1	1	1	0
$p(x_{1})$	1	0.75	0.75	0.50	0.25
$p(x_{2})$	0	0.25	0.25	0.50	0.75
$H(x_{1})$	$4\cdot log_{2}\left(1\right)$	$3\cdot \frac{3}{4}log_{2}\left(\frac{3}{4}\right)$	$3\cdot \frac{3}{4}log_{2}\left(\frac{3}{4}\right)$	$2\cdot \frac{1}{2}log_{2}\left(\frac{1}{2}\right)$	$\frac{1}{4}log_{2}\left(\frac{1}{4}\right)$
$H(x_{2})$	0	$\frac{1}{4}log_{2}\left(\frac{1}{4}\right)$	$\frac{1}{4}log_{2}\left(\frac{1}{4}\right)$	$2\cdot \frac{1}{2}log_{2}\left(\frac{1}{2}\right)$	$3\cdot \frac{3}{4}log_{2}\left(\frac{3}{4}\right)$

**Table 6:** Comparison of checked subkeys between MASA, NGA and differential cryptanalysis attacks.

Attack	Total Number of Checked Subkeys	Average Number of Checked Subkeys
MASA algorithm		
$\Omega_{1}$	687,752	22,925.1
$\Omega_{2}$	687,788	22,926.3
∑	1,375,540	45,851.3
NGA algorithm		
$\Omega_{1}$	252,456	8415.2
$\Omega_{2}$	252,899	8430.0
∑	505,355	16,845.2
Differential Cryptanalysis		
$\Omega_{1}$	$30\cdot (6\cdot 2^{30}+1024)$	$6\cdot 2^{30}+1024$
$\Omega_{2}$	$30\cdot (6\cdot 2^{30}+1024)$	$6\cdot 2^{30}+1024$
∑	$30\cdot (12\cdot 2^{30}+1024)$	$12\cdot 2^{30}+1024$

## Data Availability

Not applicable.

## Abbreviations

The following abbreviations are used in this paper:

ACO	Ant Colony Optimization
BCSO	Binary Cat Swarm Optimization
DC	Differential Cryptanalysis
DES	Data Encryption Standard
DSA	Dolphin Swarm Algorithm
EA	Evolutionary Algorithms
FEAL	Fast Data Encipherment Algorithm
GSO	Genetic Swarm Optimization
MA	Memetic Algorithms
MASA	Memetic Algorithm Simmulated Annealing
NGA	Negation Genetic Algorithms
PSO	Particle Swarm Optimization
RC4	Rivest Cipher 4
SDES	Simplified Data Encryption Standard
TEA	Tiny Encryption Algorithm
VMPC	Variably Modified Permutation Composition

## Appendix A. The Comparative and Entropy Studies for the $\Omega_{2}$ Characteristic

**Table A1:** Fitness function values for MASA and NGA algorithms for characteristic $\Omega_{P}^{2}$.

ID	MASA					NGA				
	Min	Med	Avg	Max	Std. Dev.	Min	Med	Avg	Max	Std. Dev.
1	906	984	1002.6	1095	57.1	987	1027	1041.5	1095	38.0
2	967	1059	1043.0	1095	42.6	**1008**	**1034**	**1043.3**	**1074**	**21.3**
3	946	973	997.8	1095	50.7	**993**	**1010**	**1025.2**	**1075**	**29.2**
4	1008	1041	1048.5	1095	29.2	**968**	**1047**	**1046.8**	**1074**	**31.5**
5	1008	1044	1045.6	1095	20.2	922	1021	1024.3	1095	52.7
6	943	989	1018.7	1095	60.7	**964**	**1044**	**1043.5**	**1074**	**35.4**
7	959	1041	1038.8	1095	40.0	888	957	994.0	1095	72.2
8	940	1011	1020.6	1095	44.0	953	1059	1036.3	1095	49.8
9	928	1028	1029.0	1095	56.6	958	1030	1031.6	1095	39.1
10	953	1011	1019.9	1095	47.3	**895**	**1059**	**1017.1**	**1074**	**64.7**
11	941	1041	1022.8	1095	50.8	958	1059	1043.7	1095	44.2
12	993	1033	1045.5	1095	30.0	**1006**	**1021**	**1036.7**	**1075**	**26.7**
13	955	1060	1039.2	1095	50.0	959	1053	1049.5	1095	38.1
14	946	1006	1012.2	1095	49.0	927	1027	1022.9	1095	59.4
15	**949**	**1021**	**1019.4**	**1053**	**27.0**	916	1034	1028.2	1095	56.3
16	**891**	**979**	**992.2**	**1075**	**58.3**	995	1068	1054.6	1095	32.5
17	897	1002	1004.4	1095	55.4	958	1054	1045.2	1095	46.4
18	**902**	**952**	**974.1**	**1075**	**53.2**	**939**	**963**	**989.1**	**1074**	**47.4**
19	969	1025	1033.3	1095	45.5	**884**	**989**	**990.6**	**1074**	**62.6**
20	950	1023	1037.6	1095	52.0	985	1027	1039.0	1095	35.3
21	899	1036	1026.9	1095	57.7	**940**	**977**	**992.1**	**1075**	**44.3**
22	1016	1032	1043.9	1095	27.2	957	1007	1021.2	1095	49.1
23	947	1036	1027.0	1095	56.6	**902**	**1021**	**1007.4**	**1039**	**38.0**
24	977	1068	1053.6	1095	39.7	**913**	**1013**	**1016.1**	**1074**	**48.6**
25	945	1021	1031.1	1095	42.9	**905**	**1028**	**1024.2**	**1075**	**53.2**
26	1011	1041	1043.7	1095	25.1	952	1031	1036.4	1095	50.1
27	937	1013	1010.0	1095	45.0	961	996	1013.6	1095	49.9
28	971	1002	1027.2	1095	52.1	**936**	**1017**	**1023.8**	**1074**	**46.6**
29	907	1038	1027.5	1095	64.6	**949**	**1018**	**1018.6**	**1075**	**32.0**
30	950	1018	1016.0	1095	50.4	949	1065	1054.8	1095	41.8

Where the red color indicates experiments when the algorithm wasn’t able to find the correct subkey and the green bars indicate are tests when the subkey was successfully guessed.

## References

[1] Schneier B. (1996). Applied Cryptography: Protocols, Algorithms, and Source Code in C.

[2] Menezes A.J., Oorschot P.C., Vanstone S.A. (1997). Handbook of Applied Cryptography.

[3] Biham E., Shamir A. (1991). Differential cryptanalysis of DES-like cryptosystems. J. Cryptol..

[4] Song J., Zhang H., Meng Q., Zhangyi W. (2007). Cryptanalysis of Four-Round DES Based on Genetic Algorithm. Wirel. Commun. Netw. Mob. Comput. IEEE.

[5] Tadros T., Hegazy A., Badr A. (2007). Genetic Algorithm for DES Cryptanalysis. Int. J. Comput. Sci. Netw. Secur..

[6] Garg P. (2009). A Comparison between Memetic algorithm and Genetic algorithm for the cryptanalysis of Simplified Data Encryption Standard algorithm. Int. J. Netw. Secur. Its Appl. (IJNSA).

[7] Hu W. (2010). Cryptanalysis of TEA using quantum-inspired genetic algorithms. J. Softw. Eng. Appl..

[8] Abd-Elmonim W.G., Ghali N.I., Hassanien A.E., Abraham A. Known-Plaintext Attack of DES16 Using Particle Swarm Optimization. Proceedings of the Third IEEE World Congress on Nature and Biologically Inspired Computing.

[9] Vimalathithan R., Valarmathi M.L. (2011). Cryptanalysis of simplified-DES using computational intelligence. WSEAS Trans. Comput..

[10] Jadon S.S., Sharma H., Kumar E., Bansal J.C., Deep K., Nagar A., Pant M., Bansal J. (2012). Application of binary particle swarm optimization in cryptanalysis of DES. Proceedings of the International Conference on Soft Computing for Problem Solving.

[11] Pandey S., Mishra M. (2012). Particle swarm optimization in cryptanalysis of DES. Int. J. Adv. Res. Comput. Eng. Technol..

[12] Ali I.K. (2013). Cryptanalysis of simple substitution ciphers using bees algorithm. J. Baghdad Coll. Econ. Sci. Univ..

[13] Boryczka U., Dworak K., Hwang D., Jung J.J., Nguyen N.T. (2014). Cryptanalysis of Transposition Cipher Using Evolutionary Algorithms. Computational Collective Intelligence. Technologies and Applications.

[14] Mekhaznia T., Menai M.E.B. (2014). Cryptanalysis of classical ciphers with ant algorithms. Int. J. Metaheuristics.

[15] Bhateja A.K., Bhateja A., Chaudhury S., Saxena P.K. (2015). Cryptanalysis of vigenere cipher using cuckoo search. Appl. Soft Comput..

[16] Jain A., Chaudhari N.S., Sabri A., Tingwen H., Weng K.L., Qingshan L. A New Heuristic Based on the Cuckoo Search for Cryptanalysis of Substitution Ciphers. Proceedings of the International Conference on Neural Information Processing.

[17] Amic S., Soyjaudah K.S., Mohabeer H., Ramsawock G. (2016). Cryptanalysis of DES16 using binary firefly algorithm. Proceedings of the 2016 IEEE International Conference on Emerging Technologies and Innovative Business Practices for the Transformation of Societies.

[18] Dworak K., Nalepa J., Boryczka U., Kawulok M., Król D., Madeyski L., Nguyen N.T. (2016). Cryptanalysis of SDES using genetic and memetic algorithms. Recent Developments in Intelligent Information and Database Systems.

[19] Dworak K., Boryczka U., Nguyen N.T., Iliadis L., Manolopoulos Y., Trawiński B. (2016). Differential Cryptanalysis of FEAL4 using Evolutionary Algorithm. Computational Collective Intelligence.

[20] Amic S., Soyjaudah K.S., Ramsawock G., Satapathy S., Bhateja V., Somanah R., Yang X.S., Senkerik R. (2019). Dolphin swarm algorithm for cryptanalysis. Information Systems Design and Intelligent Applications.

[21] Jain A., Chaudhari N.S. (2017). A novel cuckoo search strategy for automated cryptanalysis: A case study on the reduced complex knapsack cryptosystem. Int. J. Syst. Assur. Eng. Manag..

[22] Dworak K., Boryczka U., Nguyen N.T., Papadopoulos G.A., Jędrzejowicz P., Trawiński B., Vossen G. (2017). Genetic Algorithm as Optimization Tool for Differential Cryptanalysis of DES6. Computational Collective Intelligence.

[23] Polak I., Boryczka M. (2018). Tabu search against permutation based stream ciphers. Int. J. Electron. Telecommun..

[24] Kamal R., Bag M., Kule M., Das A., Nayak J., Naik B., Pati S., Pelusi D. (2019). On the cryptanalysis of SDES using binary cuckoo search algorithm. Computational Intelligence in Pattern Recognition.

[25] Polak I., Boryczka M. (2019). Tabu Search in revealing the internal state of RC4+ cipher. Appl. Soft Comput..

[26] Sabonchi A.K.S., Akay B. (2020). Cryptanalysis of Polyalphabetic Cipher Using Differential Evolution Algorithm. Tehnički Vjesnik.

[27] Grari H., Lamzabi S., Azouaoui A., Zine-Dine K. (2021). Cryptanalysis of Merkle-Hellman cipher using ant colony optimization. IAES Int. J. Artif. Intell..

[28] Amic S., Soyjaudah K.S., Ramsawock G. (2017). Binary cat swarm optimization for cryptanalysis. Proceedings of the 2017 IEEE International Conference on Advanced Networks and Telecommunications Systems (ANTS).

[29] Pieprzyk J., Hardjono T., Seberry J. (2003). Fundamentals of Computer Security.

[30] Stallings W. (2011). Cryptography and Network Security: Principles and Practice.

[31] Stinson D.R. (1995). Cryptography: Theory and Practice.

[32] Stamp M., Low R.M. (2007). Applied Cryptanalysis. Breaking Ciphers in the Real World.

